# Supplementary material for: AI-enhanced marker-assisted selection concept for the multifunctional honey bee (Hymenoptera: Apidea) protein Vitellogenin (Vg)
Source: J Econ Entomol. 2025 Aug 22;118(5):2133–44. doi: 10.1093/jee/toaf187 (PMC12534086; doi:10.1093/jee/toaf187)
Supplement: toaf187_Supplementary_Data [file toaf187_supplementary_data.pdf]

```
                           369
Vg_Exon2(ID:_406088)       cggggacagtttcagccgacttccagcacaattggcaagtcgggaatgagtacacgtatctcgttcgaagtcgcacgttgacgagtctgggcgatctgtcggacgtgcataccggtattctgataaaggcgttgctcaccgtacag
Exon_2_hap_1_(20_drones)   cggggacagtttcagccgacttccagcacaattggcaagtcgggaatgagtacacgtatctcgttcgaagtcgcacgttgacgagtctgggcgatctgtcggacgtgcataccggtattctgataaaggcgttgctcaccgtacag
Exon_2_hap_2_(17_drones)   cggggacagtttcagccgacttccagcacaattggcaagtcgggaatgagtacacgtatctcgttcgaagtcgcacgttgacgagtctgggcgatctgtcggacgtgcataccggtattctgataaaggcgttgctcaccgtacag
Exon_2_hap_3_(28_drones)   cggggacagtttcagccgacttccagcacaattggcaagtcgggaatgagtacacgtatctcgttcgaagtcgcacgttgacgagtctgggcgatctgtcggacgtgcataccggtattctgataaaggcgttgctcaccgtacag
Exon_2_hap_4_(13_drones)   cggggacagtttcagccgacttccagcacaattggcaagtcgggaatgagtacacgtatctcgttcgaagtcgcacgttgacgagtctgggcgatctgtcggacgtgcataccggtattctgataaaggcgttgctcaccgtacag
Exon_2_hap_5_(3_drones)    cggggacagtttcagccgacttccagcacaattggcaagtcgggaatgagtacacgtatctcgttcgaagtcgcacgttgacgagtctgggcgatctgtcggacgtgcataccggtattctgataaaggcgttgctcaccgtacag
Exon_2_hap_6_(30_drones)   cggggacagtttcagccgacttccagcacaattggcaagtcgggaatgagtacacgtatctcgttcgaagtcgcacgttgacgagtctgggcgatctgtcggacgtgcataccggtattctgataaaggcgttgctcaccgtacag
Exon_2_hap_7_(30_drones)   cggggacagtttcagccgacttccagcacaattggcaagtcgggaatgagtacacgtatctcgttcgaagtcgcacgttgacgagtctgggcgatctgtcggacgtgcataccggtattctgataaaggcgttgctcaccgtacag

                                      526                                             574
Vg_Exon2(ID:_406088)       gcgaaggattcaaacgtgcttgccgccaaagtgtggaacggccaatatgctcgtgtccaacagtccatgcctgacggatgggaaacggagatctccgaccaaatgttggagctccgtgacctgcctatttccggcaaaccgttcca
Exon_2_hap_1_(20_drones)   gcgaaggattcaaacgtgcttgccgccaaagtgtggaacggccaatatgctcgtgtccaacagtccatgcctgacggatgggaaacggagatctccgaccaaatgttggagctccgtgacctgcctatttccggcaaaccgttcca
Exon_2_hap_2_(17_drones)   gcgaaggattcgaacgtgcttgccgccaaagtgtggaacggccaatatgctcgtgtccagcagtccatgcctgacggatgggaaacggagatctccgaccaaatgttggagctccgtgacctgcctatttccggcaaaccgttcca
Exon_2_hap_3_(28_drones)   gcgaaggattcgaacgtgcttgccgccaaagtgtggaacggccaatatgctcgtgtccagcagtccatgcctgacggatgggaaacggagatctccgaccaaatgttggagctccgtgacctgcctatttccggcaaaccgttcca
Exon_2_hap_4_(13_drones)   gcgaaggattcgaacgtgcttgccgccaaagtgtggaacggccaatatgctcgtgtccagcagtccatgcctgacggatgggaaacggagatctccgaccaaatgttggagctccgtgacctgcctatttccggcaaaccgttcca
Exon_2_hap_5_(3_drones)    gcgaaggattcgaacgtgcttgccgccaaagtgtggaacggccaatatgctcgtgtccagcagtccatgcctgacggatgggaaacggagatctccgaccaaatgttggagctccgtgacctgcctatttccggcaaaccgttcca
Exon_2_hap_6_(30_drones)   gcgaaggattcaaacgtgcttgccgccaaagtgtggaacggccaatatgctcgtgtccaacagtccatgcctgacggatgggaaacggagatctccgaccaaatgttggagctccgtgacctgcctatttccggcaaaccgttcca
Exon_2_hap_7_(30_drones)   gcgaaggattcaaacgtgcttgccgccaaagtgtggaacggccaatatgctcgtgtccaacagtccatgcctgacggatgggaaacggagatctccgaccaaatgttggagctccgtgacctgcctatttccggcaaaccgttcca

                                                                  700           714                                                                 773
Vg_Exon2(ID:_406088)       gatcaggatgaaacacggtttgatcagggatctgatcgttgatcgcgacgtccccacttgggaggtgaacatactcaagagcatcgttggccaactgcaagtggacactcaaagtgaaaacgccgtgaaggtgaacagcgtgcaag
Exon_2_hap_1_(20_drones)   gatcaggatgaaacacggtttgatcagggatctgatcgttgatcgcgacgtccccacttgggaggtgaacatactcaagagcatcgttggccaactgcaagtggacactcaaagtgaaaacgccgtgaaggtgaacagcgtgcaag
Exon_2_hap_2_(17_drones)   gatcaggatgaaacacggtttgatcagggatctgatcgtggatcgcgacgtccccacttgggaggtgaacatactcaagagcatcgttggccaactgcaagtggacactcaaggtgaaaacgccgtgaaggtgaacagcgtgcaag
Exon_2_hap_3_(28_drones)   gatcaggatgaaacacggtttgatcagggatctgatcgttgatcgcgacgtcctcacttgggaggtgaacatactcaagagcatcgttggccaactgcaagtggacactcaaggtgaaaacgccgtgaaggtgaacagcgtgcaag
Exon_2_hap_4_(13_drones)   gatcaggatgaaacacggtttgatcagggatctgatcgttgatcgcgacgtccccacttgggaggtgaacatactcaagagcatcgttggccaactgcaagtggacactcaaggtgaaaacgccgtgaaggtgaacagcgtgcaag
Exon_2_hap_5_(3_drones)    gatcaggatgaaacacggtttgatcagggatctgatcgttgatcgcgacgtccccacttgggaggtgaacatactcaagagcatcgttggccaactgcaagtggacactcaaggtgaaaacgccgtgaaggtgaacagcgtgcaag
Exon_2_hap_6_(30_drones)   gatcaggatgaaacacggtttgatcagggatctgatcgttgatcgcgacgtccccacttgggaggtgaacatactcaagagcatcgttggccaactgcaagtggacactcaaggtgaaaacgccgtgaaggtgaacagcgtgcaag
Exon_2_hap_7_(30_drones)   gatcaggatgaaacacggtttgatcagggatctgatcgttgatcgcgacgtccccacttgggaggtgaacatactcaagagcatcgttggccaactgcaagtggacactcaaggtgaaaacgccgtgaaggtgaacagcgtgcaag

                                                                                                                                                    946
Vg_Exon2(ID:_406088)       ttccgaccgacgacgagccgtacgcctcgttcaaagccatggaggactccgtgggagggaaatgcgaggttctctacgatatagcgcctttgtcggactttgtgatccacagatcgccggaattggtgccgatgccgacgttgaag
Exon_2_hap_1_(20_drones)   ttccgaccgacgacgagccgtacgcctcgttcaaagccatggaggactccgtgggagggaaatgcgaggttctctacgatatagcgcctttgtcggactttgtgatccacagatcgccggaattggtgccgatgccgacgttgaag
Exon_2_hap_2_(17_drones)   ttccgaccgacgacgagccgtacgcctcgttcaaagccatggaggactccgtgggagggaaatgcgaggttctctacgatatagcgcctttgtcggactttgtgatccacagatcgccggaattggtgccgatgccgacgttgaag
Exon_2_hap_3_(28_drones)   ttccgaccgacgacgagccgtacgcctcgttcaaagccatggaggactccgtgggagggaaatgcgaggttctctacgatatagcgcctttgtcggactttgtgatccacagatcgccggaattggtgccgatgccgacgttgaag
Exon_2_hap_4_(13_drones)   ttccgaccgacgacgagccgtacgcctcgttcaaagccatggaggactccgtgggagggaaatgcgaggttctctacgatatagcgcctttgtcggactttgtgatccacagatcgccggaattggtgccgatgccgacttttgaag
Exon_2_hap_5_(3_drones)    ttccgaccgacgacgagccgtacgcctcgttcaaagccatggaggactccgtgggagggaaatgcgaggttctctacgatatagcgcctttgtcggactttgtgatccacagatcgccggaattggtgccgatgccgacgttgaag
Exon_2_hap_6_(30_drones)   ttccgaccgacgacgagccgtacgcctcgttcaaagccatggaggactccgtgggagggaaatgcgaggttctctacgatatagcgcctttgtcggactttgtgatccacagatcgccggaattggtgccgatgccgacgttgaag
Exon_2_hap_7_(30_drones)   ttccgaccgacgacgagccgtacgcctcgttcaaagccatggaggactccgtgggagggaaatgcgaggttctctacgatatagcgcctttgtcggactttgtgatccacagatcgccggaattggtgccgatgccgacgttgaag

                                      964                             997                                      1039                                      1087
Vg_Exon2(ID:_406088)       ggcgatggccgccacatggaggtgatcaagatcaagaacttcgataactgcgatcaaaggataaattatcatttcggtatgacggataactcgaggttggaacctggaacgaacaagaatggaaagttcttctcg
Exon_2_hap_1_(20_drones)   ggcgatggccgccacatggaggtgatcaagatcaagaacttcgataactgcgatcaaaggataaattatcatttcggtatgacggataactcgaggttggaacctggaacgaacaagaatggaaagttcttctcg
Exon_2_hap_2_(17_drones)   ggcgatggccgccacatggaggtgatcaagatcaagaacttcgacaactgcgatcaaaggataaattatcatttcggtatgacggacaactcgaggttggaacctggaacgaacaagaatggaaagttcttctcg
Exon_2_hap_3_(28_drones)   ggcgatggccgtcacatggaggtgatcaagatcaagaacttcgacaactgcgatcaaaggataaattatcatttcggtatgacggacaactcgaggttggaacctggaacgaacaagaatggaaagttcttctcg
Exon_2_hap_4_(13_drones)   ggcgatggccgccacatggaggtgatcaagatcaagaacttcgataactgcgatcaaaggataaattatcatttcggtatgacggataactcgaggttggaacctggaacgaacaagaatggaaagttcttctcg
Exon_2_hap_5_(3_drones)    ggcgatggccgccacatggaggtgatcaagatcaagaacttcgataactgcgatcaaaggataaattatcatttcggtatgacggataactcgaggttggaacctggaacgaacaagaatggaaagttcttctcg
Exon_2_hap_6_(30_drones)   ggcgatggccgccacatggaggtgatcaagatcaagaacttcgataactgcgatcaaaggataaattatcatttcggtatgacggataactcgaggttggaacctggaacgaacaagaatggaaagttcttctcg
Exon_2_hap_7_(30_drones)   ggcgatggccgtcacatggaggtgatcaagatcaagaacttcgacaactgcgatcaaaggataaattatcatttcggtatgacggacaactcgaggttggaacctggaacgaacaagaatggaaagttcttctcg
```

Figure S1: Exon 2 of Vg (line 1, nucleotide 369-1087) aligned to the unique haplotypes of exon 2 identified in the Pol-line drones (hap 1-7 on line 2-8). The 9 identified SNPs are in bold, and the reference nucleotide is marked in green, while the alternative nucleotide is marked in yellow.

```
                        2085                                        2894                             2927              2945
Vg_Exon_4_(ID:406088)    gtgaaccaccttctcacgcatcacgaatacgactacgaactctcaaggggatacatcgacgagaagattttggagaaccagaacatcatcacccatatgatcctcaattacgtgggaagcgaagacagcgtgatcccgcgcatcct
Exon_4_hap_1_(74_drones) gtgaaccaccttctcacgcatcacgaatacgactacgaactctcaaggggatacatcgacgagaagattttggagaaccagaacatcatcacccatatgatcctcaattacgtgggaagcgaagacagcgtgatcccgcgcatcct
Exon_4_hap_2_(17_drones) gtgaaccaccttctcacgcatcacgaatacgactacgaactctcaaggggatacatcgacgagaagattttggagaaccagaacatcatcacccacatgatcctcaattacgtgggaagcgaagacagcgtgatcccgcgcatcct
Exon_4_hap_3_(48_drones) gtgaaccaccttctcacgcatcacgaatacgactacgaactctctcggggatacatcgacgagaagattttggagaatcagaacatcatcacccacatgatcctcaattacgtgggaagcgaagacagcgtgatcccgcgcatcct
Exon_4_hap_3_(2_drones)  gtgaaccaccttctcacgcatcacgaatacgactacgaactctctcggggatacatcgacgagaagattttggagaatcagaacatcatcacccacatgatcctcaattacgtgggaagcgaagacagcgtgatcccgcgcatcct

Vg_Exon_4_(ID:406088)    ctaccttacctggtactcctccaacggcgacataaaagtaccttccaccaaagtgctagccatgatctcgagcgtgaaatcattcatggagttgagcctgaggagcgtgaaggaccgagaaacgattatttcggcggccgagaaga
Exon_4_hap_1_(74_drones) ctaccttacctggtactcctccaacggcgacataaaagtaccttccaccaaagtgctagccatgatctcgagcgtgaaatcattcatggagttgagcctgaggagcgtgaaggaccgagaaacgattatttcggcggccgagaaga
Exon_4_hap_2_(17_drones) ctaccttacctggtactcctccaacggcgacataaaagtaccttccaccaaagtgctagccatgatctcgagcgtgaaatcattcatggagttgagcctgaggagcgtgaaggaccgagaaacgattatttcggcggccgagaaga
Exon_4_hap_3_(48_drones) ctaccttacctggtactcctccaacggcgacataaaagtaccttccaccaaagtgctagccatgatctcgagcgtgaaatcattcatggagttgagcctgaggagcgtgaaggaccgagaaacgattatttcggcggccgagaaga
Exon_4_hap_3_(2_drones)  ctaccttacctggtactcctccaacggcgacataaaagtaccttccaccaaagtgctagccatgatctcgagcgtgaaatcattcatggagttgagcctgaggagcgtgaaggaccgagaaacgattatttcggcggccgagaaga

                                                                                           3209     3218                    3242                  3266
Vg_Exon_4_(ID:406088)    tcgccgaggagttgaagatcgtccccgaagagctcgttcctctggaaggaaacttgatgataaacaacaaatatgcgttgaaattcttccccttcgataaacacattctcgacaaattacccacg
Exon_4_hap_1_(74_drones) tcgccgaggagttgaagatcgtccccgaagagctcgttcctctggaaggaaacttgatgataaacaacaaatatgcgttgaaattcttccccttcgataaacacattctcgacaaattacccacg
Exon_4_hap_2_(17_drones) tcgccgaggagttgaagatcgtccccgaagagctcgttcctctggaaggaaacttgatgataaacaacaaatatgcgttgaaattcttccccttcgataagcacattctcgacaaattacccacg
Exon_4_hap_3_(48_drones) tcgccgaggagttgaagatcgtccccgaagagctcgttcctctggaaggaaacttgatgataaacaataaatatgccttgaaattcttccccttcgataagcacattctcgacaaattacccacg
Exon_4_hap_3_(2_drones)  tcgccgaggagttgaagatcgtccccgaagagctcgttcctctggaaggaaacttgatgataaacaataaatatgccttgaaattcttccccttcgataagcacattctcgacaaattacccacg
```

Figure S2: The middle and end part of exon 4 of Vg (line 1, nucleotide 2085-3266) aligned to the unique haplotypes of the same section in exon 4 identified in the Pol-line drones (hap 1-3 on line 2-5). The 6 identified SNPs are in bold, and the reference nucleotide is marked in green, while the alternative nucleotide is marked in yellow. Haplotype 3 was found for 50 drones in total; 48 represent Vg variant 15, while 2 represent Vg variant 7 (line 4 and 5, respectively).

Table S1 **The 26 Vg variants**

Vg variants	Number of drones	nsSNPs													
1	24	no nsSNPs													
2	22	p.S146G													
3	1	p.S146G	p.S154R												
4	1	p.S146G	p.S154E												
5	7	p.S146G	p.S1110T												
6	4	p.S146G	p.V1451A	p.T1503A											
7	2	p.S146G	p.S1110T	p.N1220S	p.R1284K										
8	5	p.S146G	p.S1110T	p.L1291I	p.R1292S										
9	1	p.S146G	p.S1110T	p.R1292S	p.V1451A										
10	45	p.T6M	p.P126L	p.S146G	p.S1110T	p.T1503A									
11	2	p.S146G	p.S1110T	p.L1291I	p.R1292S	p.V1451A									
12	15	p.S146G	p.S1110T	p.L1291I	p.R1292S	p.V1451A	p.T1503A								
13	4	p.S146G	p.S1110T	p.N1220S	p.R1284K	p.I1398V	p.V1451A	p.T1503A							
14	21	p.S146G	p.S1110T	p.R1284K	p.I1398V	p.V1451A	p.T1503A	p.I1536V							
15	92	p.S146G	p.S1110T	p.N1220S	p.R1284K	p.I1398V	p.V1451A	p.T1503A	p.I1536V						
16	6	p.S146G	p.S1110T	p.N1220S	p.L1291I	p.R1292S	p.V1451A	p.T1503A	p.I1536V						
17	21	p.S1110T	p.V1199I	p.N1220S	p.R1284K	p.I1398V	p.V1451A	p.T1503A	p.I1536V						
18	5	p.S15A	p.G25E	p.S146G	p.N326S	p.S1110T	p.N1220S	p.I1398V	p.T1503A	p.L1751F					
19	17	p.S146G	p.S375L	p.T522I	p.V538I	p.S1110T	p.N1220S	p.I1398V	p.V1451A	p.T1503A					
20	2	p.S146G	p.S803N	p.S1110T	p.N1220S	p.R1284K	p.I1398V	p.V1451A	p.T1503A	p.I1536V					
21	2	p.S146G	p.S1110T	p.R1174K	p.N1220S	p.R1284K	p.I1398V	p.V1451A	p.T1503A	p.I1536V					
22	11	p.S146G	p.S1110T	p.N1220S	p.R1284K	p.I1398V	p.V1451A	p.T1503A	p.I1536V	p.T1676S					
23	4	p.S146G	p.S1110T	p.M1159I	p.N1220S	p.L1291I	p.R1292S	p.I1398V	p.V1451A	p.T1503A	p.I1536V				
24	1	p.S146G	p.S1110T	p.R1174K	p.N1220S	p.R1284K	p.V1451A	p.T1503A	p.I1536V	p.T1676S	p.G1678S				
25	11	p.S146G	p.S1110T	p.N1220S	p.R1284K	p.I1398V	p.V1451A	p.T1503A	p.I1536V	p.T1676S	p.G1678S				
26	4	p.S15A	p.S146G	p.T311M	p.D608E	p.V952I	p.S1110T	p.N1220S	p.V1311M	p.T1503A	p.V1510L	p.I1536V	p.V1610M	p.L1670S	p.T1716M
Total:	**330**														
